# Connected Traffic Data Ontology (CTDO) for Intelligent Urban Traffic Systems Focused on Connected (Semi) Autonomous Vehicles

**DOI:** 10.3390/s20102961

**Published:** 2020-05-23

**Authors:** Miloš Viktorović, Dujuan Yang, Bauke de Vries

**Affiliations:** Information Systems in the Built Environment (ISBE), Department of the Built Environment, Technical University of Eindhoven, 5612 AZ Eindhoven, The Netherlands; d.yang@tue.nl (D.Y.); b.d.vries@tue.nl (B.d.V.)

**Keywords:** CTDO, ontology, connected autonomous vehicles, linked data, semantic web, SOSA

## Abstract

For autonomous vehicles (AV), the ability to share information about their surroundings is crucial. With Level 4 and 5 autonomy in sight, solving the challenge of organization and efficient storing of data, coming from these connected platforms, becomes paramount. Research done up to now has been mostly focused on communication and network layers of V2X (Vehicle-to-Everything) data sharing. However, there is a gap when it comes to the data layer. Limited attention has been paid to the ontology development in the automotive domain. More specifically, the way to integrate sensor data and geospatial data efficiently is missing. Therefore, we proposed to develop a new Connected Traffic Data Ontology (CTDO) on the foundations of Sensor, Observation, Sample, and Actuator (SOSA) ontology, to provide a more suitable ontology for large volumes of time-sensitive data coming from multi-sensory platforms, like connected vehicles, as the first step in closing the existing research gap. Additionally, as this research aims to further extend the CTDO in the future, a possible way to map to the CTDO with ontologies that represent road infrastructure has been presented. Finally, new CTDO ontology was benchmarked against SOSA, and better memory performance and query execution speeds have been confirmed.

## 1. Introduction

The predictions from the automotive industry stated that L4 (Level 4—High Automation) Autonomous Vehicle (AV) tech, according to the SAE international standardization [1], will appear in 2020 and 2021 [2]. L5 (Level 5—full Autonomy) vehicles will take more time and, therefore, predictions are not as consistent as for L4 AVs [3,4]. However, until recently, vehicles with Vehicle-to-Vehicle (V2V) and Vehicle-to-Everything (V2X) communication capabilities were mostly part of research or pilot programs. In 2019, the first mass vehicle model equipped with V2X communication capabilities based on the European Telecommunications Standards Institute (ETSI) ITS-G5 standard came out of the factories [5]. These vehicles are equipped to support “Day 1 service” capabilities [6], which are focusing on “Awareness Driving via status data” [7]. This means that, for now, these services are intended to provide informative data to connected vehicles. They aimed to achieve “Day 2” and “Day 3+” services, which enable sensing and cooperative driving [7], aiming to support L4 and L5 vehicles. 

However, in the literature that deals with L4 and L5 vehicles and their information sharing, until now, the primary focus of research and development has been on the communication and network layer of V2V and V2X communication [8]. Only limited attention has been paid to the data layer and how to efficiently integrate data that is already available within the mobility ecosystem, in order to improve the accuracy of understanding the surrounding environment. In addition, it is a common understanding that these L4 and L5 vehicles will generate great amounts of data. Intel forecasted, back in 2016, that single Connected Autonomous Vehicle (CAV) in use will stream around 500 GB of data every hour [9]. This de-facto means that in not so distant future traffic systems will face a huge amount of data coming from connected vehicles. It would also open many issues, such as memory, and vehicle data time-sensitivity. 

To overcome these obstacles for the data layer, developing a linked data graph model that can facilitate information sharing between (semi) autonomous vehicles and the surrounding built environment as a set of classes, relations and axioms, seems to be a possible solution. More specifically, this would entail the development of an ontology, as the formalized structure that defines how relationships between objects of linked data. 

The idea has been confirmed by recent publications, which also show that some ontologies have been developed in this domain [10,11]. As this approach requires integrating cross-domain data, it is important to follow at least general guidelines in developing such an ontology. Although there is no standardized methodology to do this as yet [12], frameworks have been proposed on how to design the most optimal ontology, in relation to the specific context [13,14]. To our best knowledge, there are few researches so far that have developed ontology-based representation of a vehicle in the traffic context, and they primarily focus on the advanced driver-assistance systems (ADAS). Approaches vary in these researches from the development of entity-based ontologies, with distinguishable contexts, to domain-specific ones for the purpose of validating ADAS control systems [15,16]. These, however, focus on a very specific set of contexts. Such developments are important, as they demonstrate the usability of SWT (Semantic Web Technologies) and linked data in time-sensitive environments like ADAS systems. An example of such a development is the set of three ontologies developed by the Toyota Technological Institute (TTI) team, with a goal of representing the specific set of scenarios in the traffic ecosystem [17]. These three core ontologies are: map ontology, control ontology, and car ontology for AVs [17]. This specific set of ontologies has been focused on providing a platform for the development of Advanced Driving Assistance Systems (ADAS) through ontology-based decision system creation. Although it entails a very detailed set of ontologies, it also shows a very context-specific and isolated structure. Therefore, it does not leave much space for integration with other ontologies. For example, the map ontology, in this context, represents a simple connected graph of road-sections, junctions and lanes. However, these do not contain any geospatial information, which leaves them not suitable for real-world use. Extending this ontology would not be wise to do, as there are already existing standardized ontologies that can represent this type of data [18,19]. For car ontology, although it could be generalized, it does not represent in a standardized structure. It severely limits the options for extending or mapping with other, standardized, ontologies, which, in turn, defeats the purpose of using linked data [10]. Although the products of the TTI research cannot be easily extended, it is an important demonstration of the capability of utilizing SWT for AVs. Researchers have also demonstrated the value of ontologies, in scenarios of vehicle sensing system failures, which are extreme cases of single-view point, therefore, strengthening the idea behind using ontologies for AV traffic context modeling even more [20].Therefore, to develop an ontology for integration of heterogeneous different-type real-world traffic ecosystem data, more general context ontologies, especially standardized ones, would be a better way to proceed [21]. 

The World Wide Web Consortium (W3C) and Open Geospatial Consortium (OGC) have already developed well-documented standards for defining sensory systems. The Semantic Sensory Network (SSN) is the most prominent and standardized one [22]. SSN has been developed to create a framework for delivering sensor data, dealing with remote-sensing, moving sensory platforms and in-situ monitoring and sensing. Based on previously developed Sensor Model Language (SensorML) [23] and Observations and Measurements (O&M) ontologies [24], it represents an evolutionary step in sensory data representation in Resource Description Framework (RDF) [23,25]. In the development of SSN, its creators have relied originally on the Stimulus Sensor Observation (SSO) pattern [26], which they later expanded into Sensor, Observation, Sample, and Actuator (SOSA) ontology [27]. This ontology has then been used as the basis for the development of many other ontologies that deal with sensory data. One of these extensions is the Vehicle Signals and Attribute Ontology (VSSo) [28], as it utilizes a Vehicle Signal Specification (VSS) taxonomy, in order to adapt the SOSA framework to the vehicle domain [29]. Both VSS and VSSo are built in such a way that they can provide cross-domain coherence in understanding vehicle specific signals [28]. However, these sensor ontologies only focus on vehicles, and do not incorporate other sensing and actuating devices, such as traffic lights, speed sensors, induction loops, variable signalization and other parts of digital road infrastructure. For the traffic ecosystem, geospatial data plays an important role as well. For Geospatial data, there are two prominent ontologies. The first one is based on the OpenStreetMap (OSM) data, which has been developed as a part of the LinkedGeoData project [18]. The other is GeoSPARQL which has been developed by OGC, with an idea to create a general standard for representing geospatial data in the Resource Description Framework (RDF). Additionally, the extension to SPARQL has also been developed, in order to facilitate the processing of geospatial data [19]. Although these two developments can both provide a way to store geo-data, only the GeoSPARQL provides the extension, for handling geospatial data [19]. Therefore, GeoSPARQL appears to be a more suitable ontology for representing geospatial data in the context of connected AVs (CAVs). So far, existing standards allow us to separately represent sensory and geospatial data [19,27]. It is possible to utilize existing ontologies to cover certain individual parts of the wider challenge of interconnecting all traffic-related data such as vehicle-specific signals [28]. However, there is no defined way of mapping and interconnecting these individual components. This poses an even greater challenge when the amount of data, its time-sensitivity and the way data is being transmitted by CAVs [30], are considered.

To contribute to the data layer development for L4/L5 AVs, this research aims to provide the framework for the integration of all data sources related to traffic, by developing a new ontology in line with the state-of-the-art method of Semantic Web technologies (SWT). Additionally, the aim is also to have the new ontology optimized for high volume, time-sensitive data. This means that the project strives for the graph structure that provides efficient information input, storage and retrieval, both in terms of memory and speed requirements. And the aim of this paper is to present the first phase of the project, which focuses on the vehicle sensory data. 

Therefore, the paper is structured in such a way that in Section 2, a methodology will be presented on how to utilize SWT and RDF [31] to provide a homogeneous machine-readable data structure, which supports the operation of CAVs within urban environments, through defining an appropriate new ontology. This newly developed ontology considers the challenges of handling large amounts of data, which occurs in a traffic ecosystem that supports cooperative driving of (semi) autonomous vehicles and the time-sensitivity of such data. To demonstrate the validity of such logic, we will compare the performance of the new ontology against the SOSA, using traffic simulator data, containing basic telemetry variables in Chapter 3. The paper is finalized with a conclusion. 

## 2. Methodology

This paper proposes utilizing Semantic Web technologies (SWT) and RDF [31] to provide a homogeneous machine-readable data structure, which supports the operation of CAVs within urban environments through defining an appropriate new ontology, under the name Connected Traffic Data Ontology (CTDO). This will be done by two main steps. Firstly, challenges of mapping vehicle sensory data to existing ontologies will be demonstrated in parallel with the proposed solutions to mitigate these challenges, using the newly proposed CDTO framework. It is described in detail in Sub-Section 2.1. Secondly, mapping will be proposed in the CTDO in Sub-Section 2.2, in order to provide the basis for the future extensions of the ontology into the domain of geospatial infrastructure data. 

### 2.1. Vehicle Data Mapping to RDF 

In the “Day2” and “Day3” scenarios, the main use case for collaborative driving is platooning [7]. In the case of platooning, car-following models are predominant algorithms for control of AVs. These models rely on the telemetry data from the leading vehicle(s) [32,33]. Therefore, in the platooning use case, it is required to efficiently record and extract telemetry data of vehicles and traffic flows. A similar thing can be observed with other vehicle control models [33]. Therefore, it is of crucial relevance to have the ability of fast telemetry information retrieval from the data model, of vehicles in the same road-section or the surrounding ones. Due to these requirements, the development of a single ontology is proposed, incorporating the principles of SSN and SOSA [22] as much as possible, while taking care of memory and query execution-time sensitivity, which are crucial for mobility systems facilitating CAVs. The reason why utilizing pure SSN [22] to represent the sensory data from the vehicle, is not optimal is the fact that vehicles send their data in message bulks, as defined by Basic Safety Messages (BSM) in SAE J2735 standard [34]. Additionally, it is important to expand the ontology, in order to provide a mapping to existing geospatial data representation ontologies, following frameworks set by GeoSPARQL [19].

Therefore, the main logic behind the new Connected Traffic Data ontology (CTDO) development is based on the idea of understanding the state of traffic within urban environments at any given moment, as that this is crucial for CAVs [35]. The newly developed ontology directly translates (sensory) data bulks, coming from CAVs as telemetry messages, into the state of each vehicle in the context of the traffic. In this study, only vehicle position, speed and acceleration are taken into account as the state of the vehicle. Of course, for CAVs, the list can be extended, according to Basic Safety Message (BMS), or even VSS taxonomy. However, for demonstration purposes, these three points are enough, in our opinion. 

**Figure 1:**
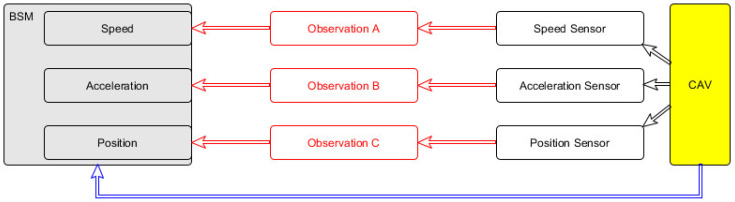
Simplified illustration of translating Basic Safety Message (BSM) data into Sensor, Observation, Sample, and Actuator ontology (SOSA)-based Resource Description Framework (RDF) graph.

An illustration of how to map BSM data into SOSA based on the RDF graph can be seen in Figure 1, where nodes and links are represented in red. It was created to accommodate part of BSM into the SOSA framework. In order to generate the proper links for mapping the BSM data to SOSA observations, certain computational power should normally be utilized, to map specific sensory observations to the responsible sensor, as this information is not carried by the BSM. For this link to be established, additional information, which is not contained in the message, has to be extracted. Therefore, extra computations have to be carried out. This is, however, not necessary in the great majority of cases, since only the results of observations and their link to the specific CAV that generated them are required. 

Additionally, multiplication of time literals for the results that are coming in bulks is also memory inefficient. Therefore, all unnecessary computational loads in generating graph elements, which are not related to CAVs are eliminated, while preserving the necessary parts of the semantic structure. That is why we propose to redefine SOSA as observation class, so that it can represent the state of the vehicle (vehicle position, speed and acceleration), or any other sensory platform, instead of a single observation.

Moreover, to represent the current state of the vehicle, and to have measurements from multiple sensors in a single observation, it is our belief that the best way to do this is to remove restriction imposed under observation module within the SOSA/SSN ontology. More specifically the restrictions:

- sosa:madeBySensor **EXACTLY** 1
- sosa:observedProperty **EXACTLY** 1

In this way, one observation would represent the state of the vehicle at one given moment. This means that the structure of SOSA/SSN in the higher levels of ontology is preserved, therefore, making it interoperable with other sensing devices in the mobility ecosystem. 

**Figure 2:**
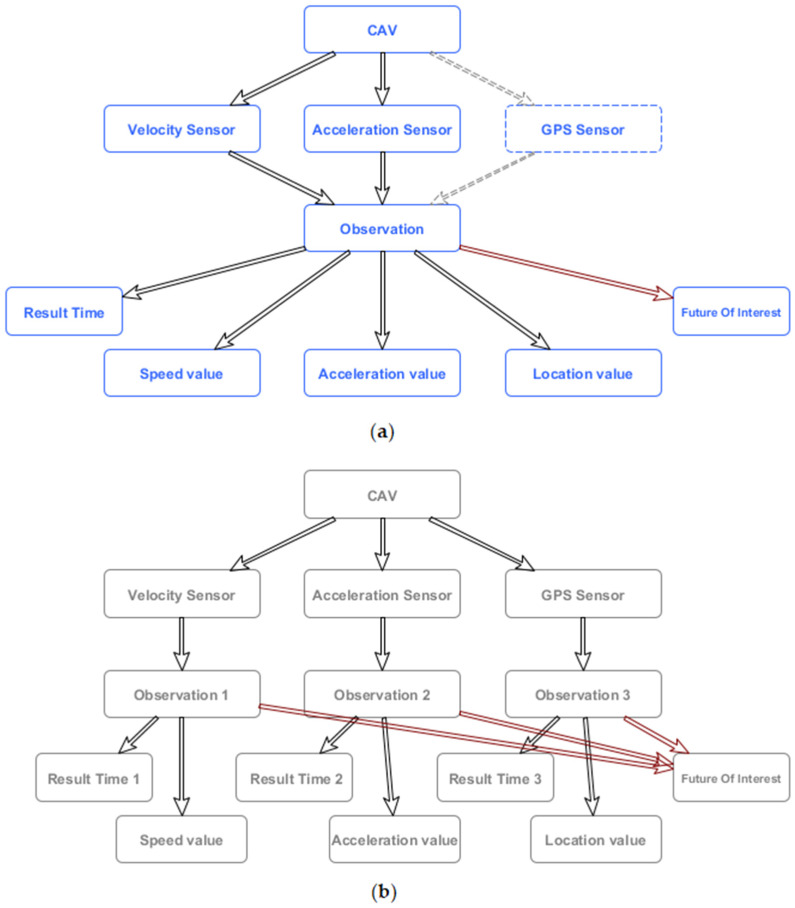
Proposed ontology (**a**) vs Sensor, Observation, Sample, and Actuator (SOSA) ontology (**b**) in simplified form.

Figure 2 shows a proposed approach, which reduces the number of connections between nodes, which, in RDF, directly translates into the number of triplets that have to be stored. If, also, in such a model, the location data would be represented as the fundamental element of the observation itself and not the observation of the specific sensor (like *sosa:resultTime*). Additionally, considering that sosa: *FutureOfInterest* node is connected to each observation, one would observe the decrease in the number of triplets $\Delta_{triplets}$ that is equal to

(1) $$\Delta_{triplets}=3n-3$$

where *n* represents the number of sensors on the given (vehicle) platform, compared to the SOSA model. 

The reason for the inclusion of the *FeatureOfInterest* node in Figure 2, is that it is the link between the observation and the actual subsystem of the general traffic ecosystem. This means that it would be possible to interconnect higher-level information in the future, like traffic intensity, to the model as well. Such higher-level information is especially interesting, as it utilizes in-vehicle hardware to provide richer information about a certain location in a certain time period. Practically, in that case, vehicles would be allowed to calculate traffic conditions at a road section they are on. This is possible, as CAVs already have preloaded geo-information data, that they utilize for navigation. Based on their speed and acceleration, they can calculate an estimate of traffic flow. This can also be achieved on the cloud, through pure GPS position information, but it is significantly more computationally demanding, when a number of cloud users are considered. As the CAVs already have certain computational capabilities, it is possible to distribute the computational load away from the centralized system, which is in accordance with IoT trends [36]. This would be important in the future because it relieves stress on the network and computational infrastructure. Therefore, it was important that this was considered and implemented in the development of the new model. 

### 2.2. Development of the New Ontology

In order to distinguish different values within the newly proposed merged observation class, properties have been created based on the properties of the SOSA model. This has been defined as sub-properties of sosa: *hasSimpleResult* property, as it follows:

#### 2.2.1. Speed

:hasSpeed
rdf:type owl:DatatypeProperty;
rdfs:domain:Vehicle_Sensor_Observation;
rdfs:range cdt:speed;
rdfs:subPropertyOf sosa:hasSimpleResult;
.

#### 2.2.2. Acceleration

:has Acceleration
rdf:type owl:DatatypeProperty;
rdfs:domain:Vehicle_Sensor_Observation;
rdfs:range cdt:acceleration;
rdfs:subPropertyOf sosa:hasSimpleResult;
.

#### 2.2.3. Road Section

To be able to connect observations to the actual road sections, the sampler module, from SOSA, had to be used as the basis. The Sample class is defined as follows: “Samples are typically subsets or extracts from the feature of interest of an observation. They are used in situations where observations cannot be made directly on the ultimate feature of interest, either because the entire feature cannot be observed, or because it is more convenient to use a proxy” [22]. That is why it has been assumed that the road section can be equivalented to the sample class of SOSA, as it represents the subset of the *FutureOfInterest* of the observation. Therefore the new sub-class of *sosa:Sample* and the new property, as a sub-property of *sosa:hasSample,* have been created, as shown below:

:RoadSection
rdf:type rdfs:Class;
rdf:type owl:Class;
rdf:type sh:NodeShape;
rdfs:label “Road section”;
rdfs:subClassOf sosa:Sample;
:hasRoadSection
rdf:type owl:ObjectProperty;
rdfs:domain sosa:FeatureOfInterest;
rdfs:range sosa:Sample;
rdfs:subPropertyOf sosa:hasSample;
owl:inverseOf:isRoadSectionOf;
.

As it can be seen in the previous code *:hasRoadSection* property also has an inverse property, which is defined as a sub-property of the inverse property of *sosa:hasSample*. 

Finally, in order to interconnect observations and samples, a sub-class of the *sosa:FeatureOfInterest* class has been defined as follows:

#### 2.2.4. Traffic Conditions

:TrafficConditions
rdf:type rdfs:Class;
rdf:type owl:Class;
rdf:type sh:NodeShape;
rdfs:comment “TrafficCondition measurements”;
rdfs:subClassOf sosa:FeatureOfInterest;
Represents traffic conditions/intensity at a certain road section, and corresponding property:
:hasTrafficCondition
rdf:type owl:ObjectProperty;
rdfs:comment “TrafficCondition measurements”;
rdfs:domain:Vehicle_Sensor_Observation;
rdfs:range:TrafficConditions;
rdfs:subPropertyOf sosa:hasFeatureOfInterest;
.

**Figure 3:**
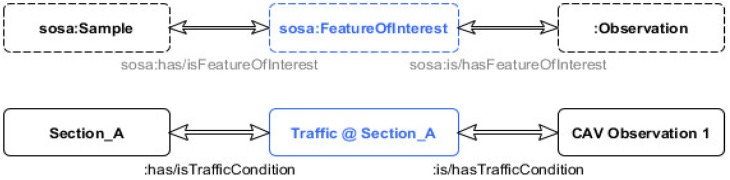
Illustration of connection between observation and road section through: TrafficCondition.

However, as the idea is to stay as close to the original SOSA framework as possible, for interoperability purposes, and yet the goal to optimize the model for data extraction purposes decision has been made to develop a more atomic structure of the *sosa:FutureOfInterest* class through its instances. That is why having an instance of *sosa:FutureOfInterest* per road section is beneficial, as the link can be directly generated between the state of the vehicle and the section that the vehicle is in. This also allows the reduction of computational time, required to generate extra elements. Additionally, this approach gives higher control in defining standardized instances of the class *TrafficConditions*, from a centralized point, rather than leaving it to the edge devices themselves. In this way, compatibility with the SOSA structure is preserved, while allowing a controlled expansion of the model. Figure 3 illustrates the practical application and connection between observations of vehicle and road section through a sub-property of *FeatureOfInterest* class from SOSA, as previously defined through the newly proposed modifications on ontology.

## 3. Validation

### 3.1. Apparatus and Data

In order to validate the performance improvement of the newly proposed model, the decision has been taken to benchmark it against the SOSA/SSN model of the same data. This is a logical step to show that the proposed modifications of SOSA have led to a more optimized model. To provide realistic data the Simulation of Urban MObility (SUMO) [37] traffic simulator data has been used. SUMO has been chosen because it represents a versatile micro-simulation platform, which can be easily extended through Python scripts. Namely, a digital model of road network surrounds the campus of the Technical University of Eindhoven, has been created. This is the area defined by the following streets in the city of Eindhoven (The Netherlands): John F. Kenedylaan, Onze Lieve Vrouwestraat, Insulindenlaan and Prof. Dr. Dorgelolaan. Then random traffic has been generated within this network, using SUMO *randomTrips()* function. Afterward, during simulation execution, through TraCI extension [38], data from vehicles in the network has been retrieved. In line with simulation execution, the SPARQL INSERT queries have been executed, with data extracted using TraCI module, in parallel to both newly proposed and the baseline SOSA/SSN models. 

On this occasion, Apache Jenna Fuseki [39] has been used, and the execution time of HTML POST requests carrying SPARQL INSERT queries has been measured. In order to limit the number of external influences on the results, a partially isolated machine has been used, and, in addition, the order of the parallel queries has been constantly changing, through the random function. Partially isolated, in this specific case, means we have eliminated all the background processes within the limits of the user privileges. This semi-isolated machine has the following specifications:

- System Model: Precision WorkStation T5500
- Processor: Intel(R) Xeon(R) CPU X5660 @ 2.80 GHz, 6 Core(s), 12 Logical Processor(s)
- Total Physical Memory: 12.0 GB
- Available Physical Memory: 7.76 GB
- Total Virtual Memory: 21.0 GB
- Available Virtual Memory: 13.0 GB
- Page File Space: 9.00 GB
- HDD Model: WDC WD3000HLFS-75G6U1

Data that has been used in this experiment represents the realistic data that is being streamed from CAVs. The chosen simulator utilized Python interface, which gave the possibility to expand it with the custom code for performing POST requests, carrying SPARQL queries. A simple insert-into string manipulation has been performed, as can be seen in Table 1, to generate these queries from the data extracted using TraCI module of SUMO. In the experiment, 4272 parallel INSERT DATA queries have been executed. Parallel execution through POST requests has been performed on the Apache Jena Fuseki server V 3.13.1. Out of 4272 requests, 100% has been successful. It is worth noting that the system was not able to cope with the speed at which data is generated in the SUMO simulator. That is why the time-break period between queries had to be forced to a minimum of 0.5 s. After performing this adaptation, the system successfully managed to execute all INSERT DATA queries, without failure. 

### 3.2. Memory Improvements

During this process, the number of triplets in both graphs was observed. Results show that, on average, per INSERT DATA query, there are 9.8 triplets in the SOSA/SSN based graph and 6.8 triplets in the graph based on our new CTDO ontology. This shows that the mathematical calculation in Equation (1) is correct. Delta here is 3, which corresponds to formula value 3n-3, as values form two sensors (speed and accele ration) have been shared. It can be stated that in the domain of storing sensor observations, reduction of the number of triplets by 30% has been achieved, in our demonstrational case.

### 3.3. Query Execution Speed Improvements

The experiment has been designed in such a way that it compared performance on both completely empty graph storage and partially filled graph storage. For the results of the empty graph, the first 50 query request execution times have been taken, while for partially filled graph 4272 queries written on top of triplets per graph, have been analyzed. As can be seen in Table 2, the difference is less than half of the millisecond in the average query request execution time. Additionally, the distribution of the results corresponds to the distribution of the larger data set. Therefore, the focus will be on analyzing a larger dataset, due to the fact we can provide more certainty having the greater number of data points.

**Figure 4:**
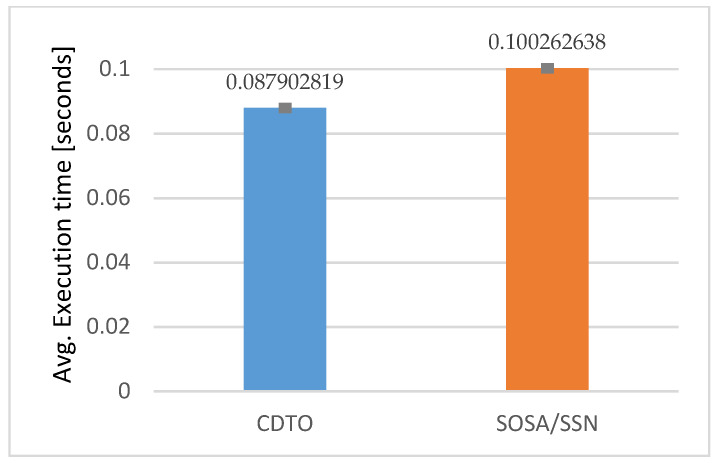
Average insert query request execution time for two different ontologies.

The aaverage execution time of the query from Table 1 on a partially filled graph (495 thousand triplets in SOSA/SSN equivalent model) was 87.90 ms (with a standard error of the mean being 0.64 ms), which is 12.36 ms better than with writing equivalent data using SOSA/SSN graph structure (which has a standard error of the mean of 0.74 ms). This represents an improvement in query execution time of more than 12%, as can be seen in Figure 4.

**Figure 5:**
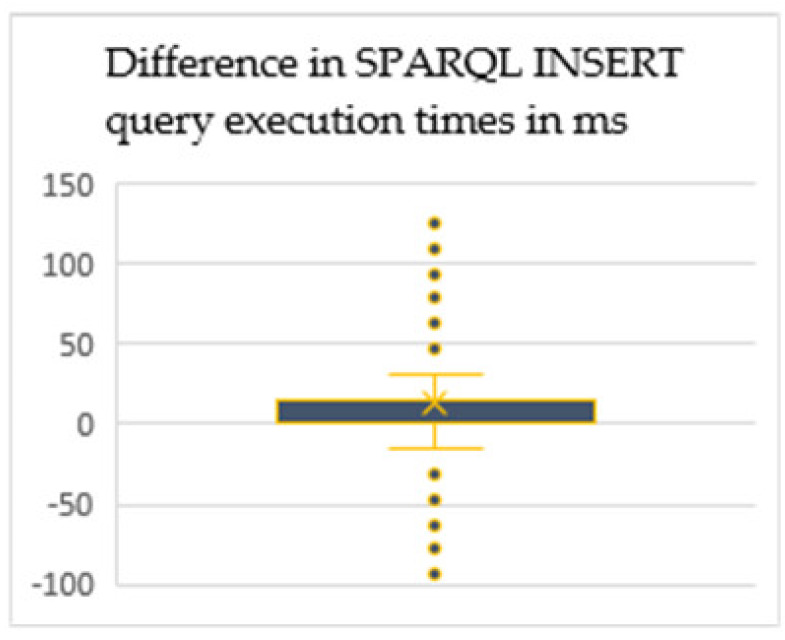
Box and whisker plot representing the difference in SPARQ INSERT DATA query request execution times distribution.

In the results obtained, the improved query response time in the newly proposed CTDO ontology has been identified, in comparison to the SSN framework. Although in the individual request executions there are outliers as seen in Figure 5, the majority of data points suggest that there is an improvement in query execution time. Namely the analysis of outliers, which accredit to around 20% of the total sample, could not identify any common cause. When repeating the same queries request, it was not able to replicate spikes in the execution time. Therefore, it can be concluded that external influences, that could not have been fully isolated on the managed machine, might have played a role in increased execution times.

**Figure 6:**
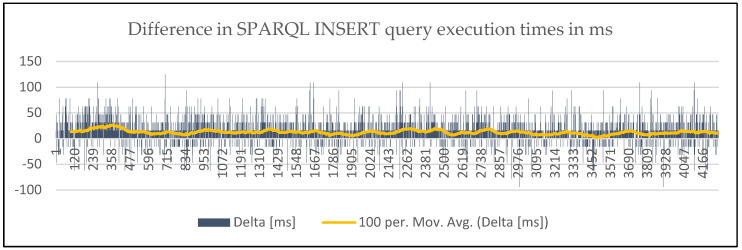
Difference in SPARQ INSERT DATA query request execution times with moving average.

This can be also seen in Figure 6, where the moving average (per 100 points) of delta in execution times, which is always above 0, can be observed. It becomes clear that there are better execution times of the INSERT query of the newly proposed ontology throughout experiments.

### 3.4. Discussion 

Overall, by defining sub-classes of SOSA classes and proposing road-section based instancing of the new sub-classes, like *sosa:FutureOfInterest*, and *sosa:Sample*, we managed to adapt the ontology to serve the mobility sector better, while maintaining full compatibility with SSN/SOSA. This has also opened a way to interconnect geospatial information and traffic infrastructure models. Incorporating such geospatial data would allow for the creation of a complete digital twin of the traffic ecosystem. Once merged data, in the form of a digital twin RDF graph, it would allow for the creation of intelligent services for vehicle and traffic control. This would then lead to the location-based services, capable of providing (automatically) enriched information, since the SWT provides a machine-readable data structure. Once these services are incorporated into CAVs, they can be utilized to mitigate the single-view point problem, and help with a better understanding of the environment the vehicle is in. However, the results also show that we cannot achieve performance and scalability required for CAVs with utilized software tools for handling RDF based data. The limitations of the used tools are such that they are not yet suitable for use in distributed and time-sensitive environments, which is shown by the results as well. That is why, in order to have the ability to utilize the advantages brought by SWT, better and more scalable tools would have to be developed, in order to be able to satisfy the response time requirements of L4 and L5 CAVs. Therefore, we aim to continue testing new tools and software solutions, while focusing on the primary research goal of further upgrading CTDO.

In future work, the CTDO will be expanded in two ways. Firstly, we intend to expand the number of defined standardized signals, led by an idea from VSSo and communication protocols like BSM. Secondly, it is planned to expand the ontology, to enable direct mapping with GeoSPARQL or similar representations of infrastructure digital twins. 

## 4. Conclusions

The need for the development of a new ontology to represent vehicles within the traffic ecosystem has been recognized, however, there is no existing ontology that can provide us with a framework to achieve this. More specifically, there are standardized ontologies for representing sensory and geospatial data, but the combination of two does not exist, especially the combination that can efficiently store the V2X broadcast messages coming from CAVs. Therefore, as a baseline, for closing this gap, a new CTDO ontology has been developed, considering the specific use cases of connected vehicles. The new CTDO ontology maintains the compatibility with SOSA as much as possible, since SOSA is a standardized framework. At the same time, it enables direct mapping between parts of BSM and CTDO by redesigning parts of the SSN/SOSA model. During this process, as the basic principles of SOSA in higher vertical layers have been maintained, CTDO can be utilized for sharing traffic-related data from any sensory device, and not only CAVs.

This has further led to significant savings in the number of RDF statements that have to be stored. It has been demonstrated that the number of triplets can be reduced by 3*n−*3, where *n* is the number of sensors on the platform. This is a substantial saving when considering a number of sensors in vehicles and the frequency at which they must stream data. Such a reduction of the number of triplets in the graph has also led to better performance in query execution times for data inserts. Additionally, a reduction in the average request time of more than 12%, in comparison to the original SOSA framework has been achieved. This has demonstrated that the newly proposed CTDO ontology is better suited for multi-sensor platforms, like connected vehicles. 

## Figures and Tables

**Table 1:** SPARQL query for data insert into Connected Traffic Data Ontology (CTDO) model.

SPARQL Query for Inserting Data into Proposed CTDO Model	Replacement %-Values (Ordered)
prefix: http://milos.viktorovic.com/CAV_V1# //CTDO prefix sosa: <http://www.w3.org/ns/sosa/> prefix cdt: <http://w3id.org/lindt/custom_datatypes#> prefix geo: <http://www.opengis.net/ont/geosparql#> prefix rdfs: <http://www.w3.org/2000/01/rdf-schema#> prefix xsd: <http://www.w3.org/2001/XMLSchema#> INSERT DATA { :Vehicle_Sensor_Observation_%s a :Vehicle_Sensor_Observation; sosa:madeBySensor :Vehicle_Sensor_%s_Speed; sosa:madeBySensor :Vehicle_Sensor_%s_Acceleration; :hasAcceleration “%f m/s2”^^cdt:acceleration; :hasSpeed “%f m/s”^^cdt:speed; :resultLocation “Point(%f %f)”^^geo:wktLiteral; sosa:observedProperty :AccelerationOfVehicle; sosa:observedProperty :SpeedOfVehicle; sosa:resultTime “%s”^^xsd:dateTime; :hasTrafficCondition :TrafficConditions_section_%s; }	(1) cav_id + ‘_’ + str(datetime), (2) cav_id,cav_id, (3) cav_id, (4) acceleration, (5) speed, (6) lon, (7) lat, (8) datetime, (9) road_section_id

**Table 2:** Comparison of the result of query execution times on the completely empty and partially filled graph.

Avg Query Execution Time in ms	CTDO	SOSA/SSN Framework	Delta
**Empty graph**	74.04	86.88	12.84
**Partially filled graph**	87.90	100.26	12.36
